# Effect of Passive Leg Raising on Systemic Hemodynamics of Pregnant Women: A Dynamic Assessment of Maternal Cardiovascular Function at 22–24 Weeks of Gestation

**DOI:** 10.1371/journal.pone.0094629

**Published:** 2014-04-14

**Authors:** Åse Vårtun, Kari Flo, Ganesh Acharya

**Affiliations:** 1 Women's Health and Perinatology Research Group, Department of Clinical Medicine, Faculty of Health Sciences, University of Tromsø and Department of Obstetrics and Gynaecology University Hospital of Northern Norway, Tromsø, Norway; 2 Department of Clinical Sciences, Intervention and Technology, Karolinska Institute, Stockholm, Sweden; Gentofte University Hospital, Denmark

## Abstract

**Objective:**

To investigate functional hemodynamic response to passive leg raising in healthy pregnant women and compare it with non-pregnant controls.

**Materials and Methods:**

This was a prospective cross-sectional study with a case-control design. A total of 108 healthy pregnant women at 22–24 weeks of gestation and 54 non-pregnant women were included. Cardiac function and systemic hemodynamics were studied at baseline and 90 seconds after passive leg raising using non-invasive impedance cardiography.

**Main outcome measures:**

Trends and magnitudes of changes in impedance cardiography derived parameters of cardiac function and systemic hemodynamics caused by passive leg raising, and preload responsiveness defined as >10% increase in stroke volume or cardiac output after passive leg raising compared to baseline.

**Results:**

The hemodynamic parameters in both pregnant and non-pregnant women changed significantly during passive leg raising compared to baseline, but the magnitude and trend of change was similar in both groups. The stroke volume increased both in pregnant (p = 0.042) and non-pregnant (p = 0.018) women, whereas the blood pressure and systemic vascular resistance decreased (p<0.001) following passive leg raising in both groups. Only 14.8% of pregnant women and 18.5% of non-pregnant women were preload responsive and the difference between groups was not significant (p = 0.705).

**Conclusion:**

Static measures of cardiovascular status are different between healthy pregnant and non-pregnant women, but the physiological response to passive leg raising is similar and not modified by pregnancy at 22–24 weeks of gestation. Whether physiological response to passive leg raising is different in earlier and later stages of pregnancy merit further investigation.

## Introduction

Static measures of cardiovascular function, such as central venous pressure, pulmonary capillary wedge pressure, mean arterial pressure, ventricular volumes etc., are poor predictors of disease severity and response to therapeutic interventions [1]. Therefore dynamic functional parameters that measure the response of the cardiovascular system to controlled variations in preload/afterload are gaining popularity in clinical practice [2], [3]. In recent years, hemodynamic response to passive leg raising (PLR) has been popularized as a dynamic test of preload responsiveness [4]. This manoeuvre provides an “auto-fluid challenge” which is rapid, transient and reversible. PLR transfers blood contained in the venous reservoir of the lower extremities to the central venous compartment leading to a transient increase in preload and an increase in cardiac output by Frank-Starling mechanism in preload responsive individuals.

Hemodynamic response to PLR has been assessed using various invasive and non-invasive techniques and it has been found to be useful in predicting fluid responsiveness in critically ill patients [5]–[7]. It has been frequently used in intensive care units to evaluate preload reserve and monitor fluid and resuscitation therapy. However, only a few small studies have evaluated hemodynamic effects of PLR in healthy subjects (mostly men) [8]–[11], and to our knowledge none in healthy pregnant women. A recent study showed that PLR maybe a useful test to guide fluid therapy in severe preeclampsia as it predicted fluid responsiveness in oliguric patients [12]. However, physiological response to PLR in normal pregnancy has not been studied yet. Pregnancy causes profound physiological changes. The circulating blood volume, heart rate, cardiac output and oxygen consumption are increased [13]–[15]. Initially there is a decrease in blood pressure (BP) and systemic vascular resistance (SVR) followed by an increase during the third trimester [16]. Cardiac morphological changes during pregnancy are characterised by reversible left ventricular hypertrophy and chamber enlargement [17], and studies on cardiac function report altered left ventricular systolic and diastolic performance [17],[18]. The cardiovascular system of the pregnant women may respond differently to a variety of challenges. For example, it is well established that physiological response to angiotensin II is blunted in pregnancy [19]. The baroreceptor reflex activity is attenuated during pregnancy [20], and an improved tolerance to orthostatic stress has been reported in conditions associated with increased circulatory volume including pregnancy [21]. Orthostatic tolerance correlates positively with plasma volume and negatively with baroreceptor activity [22]. Therefore, the magnitude and character of cardiovascular response to PLR may be different in pregnancy compared with the non-pregnant state. However, whether pregnancy modifies cardiovascular response to a transient increase in preload caused by PLR is not known.

In this study we tested the null hypothesis that non-invasively assessed preload reserve is not different between pregnant and non-pregnant women, and they are equally preload responsive. Our objective was to investigate functional hemodynamic response to PLR in healthy pregnant women at 22–24 weeks of gestation and compare it with non-pregnant controls. We chose this gestation because placental circulation is fully established by this time in pregnancy with resulting cardiovascular adaptive changes.

## Methods

**Figure 1:**
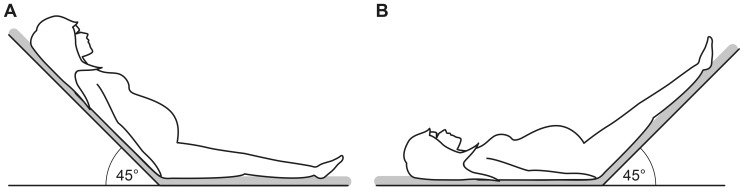
Positioning of the study participant for functional hemodynamic evaluation. Hemodynamic measurements were obtained using impedance cardiography with the women in a supine semi-recumbent position (A) after 10 minutes of rest (baseline) and 90 seconds after passively elevating both legs to 45° with the head and trunk lowered to the supine position (B) using an electronically pivotable bed.

This was a prospective cross-sectional study with a case-control design. The study was approved by the Regional Committee for Medical and Health Research Ethics - North Norway (Ref.nr. 5.2005.1386. Date of approval: 12.03.2010). Written, informed consent was taken of the study participants. A total of 108 low-risk pregnant women and 54 healthy non-pregnant controls, aged >18 years, participated in the study. Pregnant women attending for the second trimester routine ultrasound screening at 17–19 weeks of gestation were informed about the study and invited to participate if they had a low-risk pregnancy and ultrasound scan did not show any fetal or placental abnormality. Those who agreed were consecutively enrolled and an appointment was made for functional hemodynamic evaluation at 22–24 weeks of gestation. Exclusion criteria were any pre-existing medical condition that may have an effect on the course of pregnancy, and a previous history of preeclampsia, gestational diabetes, intrauterine fetal growth restriction or preterm delivery. Non-pregnant controls were recruited among the nursing, administrative and laboratory staff of the hospital and university. Healthy women of reproductive age were asked to attend for hemodynamic assessment during the follicular phase between day 5 and 10 of the menstrual cycle. Women with a previous history of pregnancy complication and those with a known disease or on regular medication were excluded. Examination was performed, in a non-fasting state between 8:30–16:00 hours in a quiet room with stable temperature. Height was measured using an altimeter (Charder Electronic Co, Taichung City, Taiwan) and weight was measured using an electronic weight (Soehnle, Leifheit AG, Nassau, Germany). Booking weight of the pregnant women was obtained from their handheld medical records. The body mass index (BMI) was calculated as weight/height^2^ using current body weight, and the body surface area (BSA) was calculated using the Du Bois formula [23]. An electronically pivotable bed designed for changing position without any active movement by the study participant was used. Hemodynamic parameters were measured using impedance cardiography (ICG) (Phillips Medical Systems, Androver, MA, USA) as described previously [24]. Baseline measurements were obtained after approximately 10 minutes of rest in a supine recumbent position with the upper part of the bed at a 45° tilt. Then the upper part of the bed was rapidly lowered to a supine position and passive raising of both legs was obtained by elevating the lower part of the bed to 45° (Figure 1). The hemodyamic measurements obtained at approximately 90 seconds after PLR were compared with the baseline values. Percent change (Δ%) in each hemodyamic parameters from baseline to PLR was calculated as: (measurement during PLR-baseline measurement)/measurement during baseline x 100. Subjects demonstrating >10% increase in stroke volume (SV) or cardiac output (CO) after PLR were considered to be preload responsive [3], [5], [25]. Information on the course and outcome of pregnancy was obtained from the electronic hospital records.

### Sample size calculation

A priori sample size calculation was performed with a desired case/control (pregnant/non-pregnant) ratio of 2∶1. For an 80% chance of detecting differences between groups at a significance level (alpha) of 0.05 assuming that approximately 25% women in the non-pregnant group and twice as much (50%) in the pregnant group would be preload responsive, we calculated a required total sample size of 150 women (100 pregnant and 50 non-pregnant) incorporating continuity correction. A total of 162 women (108 pregnant and 54 non-pregnant) were recruited to account for any possible measurement failures, dropouts and loss to follow-up.

### Statistical methods

Data were analysed using IBM SPSS statistics 20.0. Continuous variables are presented as mean (±SD) or median (range) and categorical variables as n (%) as appropriate. Differences between pregnant and non-pregnant groups were analysed using independent sample t-test for parametric continuous variables and chi-squared test for categorical variables. The hemodynamic variables measured at baseline and 90 seconds after PLR within each group were compared using paired-sample t-test. A two-sided p-value of <0.05 was considered significant.

## Results

The characteristics of the study population are presented in Table 1. There were no significant differences in age and previous obstetric history between two groups, but as expected, the pregnant women had significantly higher BMI and lower mean arterial blood pressure (MAP). The mean booking weight of the pregnant women was 67.91±13.55 Kg, which was approximately 5 Kg lower than the mean current weight.

**Table 1:** Baseline characteristics of the study population.

Variable	Pregnant	Non-pregnant	p - value
Age (years)	30 (19–39)	30 (20–39)	0.481
Body weight (Kg)	72.99±13.07	68.06±10.93	0.018
Height (m)	1.67±0.06	1.69±0.06	0.117
Body mass index (Kg/m^2^)	26.09±4.18	23.88±3.54	0.001
Body surface area (m^2^)	1.81±0.16	1.77±0.14	0.136
Nulliparous	63 (58.3)	29 (53.7)	0.154
Mean arterial pressure (mmHg)	78.88±6.45	84.72±7.73	<0.001

Data presented as n (%), median (range) or mean ± SD as appropriate.

None of the pregnant women developed any significant pregnancy complications. The mean gestational age at delivery was 40 (range, 36–42) weeks. Two women delivered at 36 weeks of gestation; they were not excluded from analysis. Thirteen women were delivered by a cesarean section. Three of them had elective cesarean section due to breech presentation and ten had an emergency cesarean section (eight for failure to progress, one for placental abruption and one for fetal distress). The mean birth weight of the infants was 3634 (±524) g and the placental weight was 628 (±129) g. The median 5-minute Apgar score was 10 (range, 7–10), the mean umbilical artery pH was 7.25 (±0.08) and the base excess −3.83 (±3.49) mmol/L.

The results of hemodynamic measurements obtained at baseline and 90 seconds after PLR are shown in Table 2. The ICG parameters describing systemic blood flow and resistance, i.e. heart rate, mean arterial pressure (MAP), cardiac index (CI), systemic vascular resistance index (SVRI) were significantly (p<0.001) different between groups at baseline. The heart rate was 21.7% higher, MAP 6.9% lower, CI 24.8% higher and SVRI 26.6% lower among pregnant women compared to non-pregnant. Among the parameters describing cardiac contractility and work, accelerated cardiac index (ACI) was 10.1% higher (p = 0.050), velocity index (VI) was 13.4% higher (p<0.001), left ventricular pre-ejection period (PEP) was 11.4% shorter (p<0.001), left ventricular ejection time (LVET) 10.0% shorter (p<0.001), systolic time ratio (STR) was similar (p = 0.934), and the left ventricular cardiac work index (LCWI) was 13.2% higher (<0.001) among pregnant compared to non-pregnant women.

**Table 2:** Hemodynamic parameters measured by impedance cardiography at baseline and 90 seconds after passive leg raising (PLR) in pregnant and non-pregnant women.

	Pregnant				Non-pregnant				p-value*
Hemodynamic parameter	Baseline	PLR	% Change	p-value#	Baseline	PLR	% Change	p-value#	
SI (ml/m^2^)	45.99±6.31	46.81±6.66	2.21±9.96	0.048	44.69±5.38	45.74±6.19	2.42±7.60	0.020	0.888
CI (L/min/m^2^)	3.62±0.55	3.62±0.55	0.49±9.85	0.955	2.90±0.47	2.94±0.52	1.34±9.08	0.334	0.596
BPS (mm Hg)	101.72±8.33	99.32±7.66	−2.22±4.62	<0.001	107.98±9.48	104.04±8.65	−3.51±4.46	<0.001	0.090
BPD (mm Hg)	67.45±6.57	63.33±5.83	−5.93±4.92	<0.001	73.09±7.79	67.91±7.10	−6.99±4.30	<0.001	0.182
SVRI (dyne s m^2^/cm^5^)	1685.74±269.04	1605.65±235.33	−4.14±9.50	<0.001	2298.15±375.30	2154.26±382.37	−6.04±9.25	<0.001	0.228
ACI (1/100 s^2^)	147.31±55.57	140.72±46.92	−1.99±22.85	0.072	133.80±31.37	128.56±38.94	−2.10±29.01	0.231	0.980
LWCI (kg m/m^2^)	3.69±0.70	3.52±0.72	−3.75±12.65	<0.001	3.26±0.70	3.09±0.72	−5.06±9.70	0.001	0.504
PEP (ms)	76.77±15.84	67.76±13.57	−10.36±14.89	<0.001	86.67±14.68	76.22±13.83	−11.66±9.37	<0.001	0.498
LVET (ms)	260.07±30.66	268.47±30.81	4.34±15.28	0.018	288.85±23.53	300.35±30.60	4.46±12.14	0.014	0.958
VI (1/1000 s)	89.70±24.23	84.04±20.19	−4.68±15.00	<0.001	79.07±16.35	76.93±19.15	−2.78±13.96	0.158	0.439
STR (%)	30.56±8.51	25.79±5.57	−12.45±19.52	<0.001	30.46±5.58	25.94±5.38	−14.08±14.65	<0.001	0.588

Data are presented as mean ± SD. % Change is the difference between values obtained at baseline and after PLR in percent.

# Represents the p-value for the difference between baseline and PLR (paired sample t-test) within pregnant and non-pregnant groups.

^*^Represents the p-value for the difference in % change between pregnant and non-pregnant groups (independent sample t-test). SI, stroke index; CI, cardiac index; BPS, systolic blood pressure; BPD, diastolic blood pressure; SVRI, systemic vascular resistance index; ACI, accelerated cardiac index; LWCI, left ventricular work index; PEP, pre-ejection period; LVET, left ventricular ejection time; VI, velocity index and STR, systolic time ratio.

**Figure 2:**
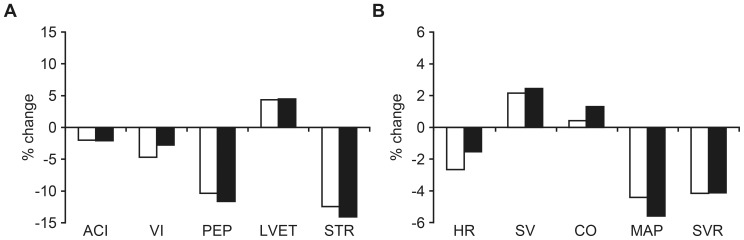
Changes in parameters of cardio-vascular function from baseline to 90 seconds after passive leg raising. A. Systolic function and contractility B. Systemic blood flow and resistance. White bars represent pregnant women and black bars represent non-pregnant women.

The majority of hemodynamic parameters changed significantly following PLR compared to baseline in both pregnant and non-pregnant women, and the trend was similar in both groups (Table 2 and Figure 2). The magnitude of change (Δ%) in ICG parameters describing cardiac systolic function/contractility, systemic blood flow and resistance are presented in Figure 2. We found a 2.65% decrease (p<0.001) in heart rate, 2.15% increase (p = 0.042) in SV, 0.43% increase (p = 0.915) in CO, 4.41% decrease (p<0.001) in MAP and 4.16% decrease (p<0.001) in SVR after PLR among pregnant women. The corresponding values for non-pregnant women were a 1.54% decrease (p = 0.120) in heart rate, 2.44% increase (p = 0.018) in SV, 1.31% increase (p = 0.295) in CO, 5.58% decrease (p<0.001) in MAP and 4.12% decrease in SVR (p<0.005), respectively. The ACI (1.99% and 2.10%), VI (4.68% and 2.78%), PEP (10.36% and 11.66%), STR (12.45% and 14.08%), LWCI (3.75% and 5.06%) decreased and the LVET (4.34% and 4.46%) increased after PLR in both pregnant and non-pregnant women, respectively. The changes were significant for PEP (p<0.001), LWCI (p<0.001) and LVET (p<0.018) in both groups, and for VI (p<0.001) only among pregnant women. The change in ACI was not statistically significant in both groups. The percent change from baseline to PLR was not significantly different between pregnant and non-pregnant women for any of the measured variables.

Only 13% of pregnant women and 18.5% of non-pregnant women increased their CO >10% following leg raising and the proportion was 14.8% and 11.1%, respectively for the SV. The differences between groups were not statistically significant.

## Discussion

Physiological adaptation is needed in pregnancy to meet the metabolic demands of mother and fetus, and as expected, there were clear differences in cardiovascular status between pregnant and non-pregnant women in our study. Transient volume load as a result of PLR led to significant hemodynamic changes in both groups, but with similar trend and magnitude suggesting that the response to mild functional hemodynamic stress is not modified by pregnancy. Previous studies have shown that increased CO in pregnancy is adequately maintained during postural changes [26] and even an acute loss of 450 ml blood does not significantly change the hemodynamic response to orthostatic stress in pregnant women [27]. The magnitude of change in SVR during orthostatic stress is reported to be greater in non-pregnant women compared to pregnant [21]. Blood volume mobilized by PLR could be larger in pregnant women as physiological pregnancy is associated with an increase in circulatory volume and venous capacitance in the lower extremities [28]. However, it did not appear to be the case as the SV and CO increased only by 1.6 mL and 0.01 L/min, respectively from the baseline to PLR in pregnant women compared to 1.9 mL and 0.07 L/min in non-pregnant, and the differences between groups were not significant.

Our study showed that normal hemodynamic response to PLR is preserved in pregnancy. As expected, PLR significantly increased SV and reduced BP and SVR in both pregnant and non-pregnant women. However, as the heart rate decreased significantly (p<0.001) by PLR in pregnant women, but insignificantly (p = 0.120) in non-pregnant, the increase in CO was insignificant in both groups. The CO is determined by heart rate, left ventricular preload, afterload and contractility. ACI (reflects peak acceleration of blood flow from the left ventricle into the aorta), VI (reflects maximum change in impedance after opening of the aortic valve and is equivalent to the maximum velocity of the systolic wave of aortic blood flow), PEP (reflects isovolemic contraction time of the left ventricle and is equivalent to electrical systole), LVET (time between the opening and closing of the aortic valve that reflects the duration of left ventricular ejection and is equivalent to mechanical systole) and STR (ratio of electrical to mechanical systole) are ICG-derived surrogate measures of cardiac systolic function and contractility. As judged by the direction and magnitude of change of these parameters (Figure 2), left ventricular contractility did not increase as a result of PLR in both study groups.

PLR is a modified Trendelenburg position that was used extensively in the initial management of hypovolemic shock until it fell into disfavour due to its small [29] and unsustained [8] effect on hemodynamics and possible adverse effects [30]. PLR augments venous return, increases central venous and pulmonary pressures and enhances cardiac preload and performance [31], [32] leading to an increase in SV and CO, but the changes are shown to be small in healthy subjects using echocardiography [10] and impedance cardiography [9]. Our study confirms that the hemodynamics changes caused by PLR are of small magnitude both in healthy pregnant and non-pregnant women.

Blood volume mobilised by leg raising can vary even among healthy individuals depending on their body composition, circulating blood volume, state of hydration etc. Amount of blood that can be mobilized into the central circulation by PLR remains controversial. Using nuclear scintigraphy, Rutlen et al [33] reported a 30–35% decrease in calf radioactivity after PLR, which corresponds to a blood volume of 150 ml transferred to the intravascular space [8]. Gaffney et al [8] measured an 8–10% increase in CO and SV following PLR. Bivins et al [29] studied blood volume distribution in 15° Trendelenburg position in 10 healthy subjects and found that it resulted in displacement of only 1.8% of total volume centrally. The increase in SV induced by PLR is larger in healthy subjects after withdrawal of 500 ml blood [34] and preload responsive volume depleted patients usually show >10–12% increase in SV [5]. This suggests that the response to PLR can be modified by central volume status and baseline preload. However, despite significantly increased plasma volume in pregnancy [14], the magnitude and character of response was not different in pregnant women compared to non-pregnant. Furthermore, less than 15% of pregnant women were found to be preload responsive at 22–24 weeks of gestation. This may be explained by the fact that pregnancy is associated with increased circulating volume and attenuated baroreflex activity, which are known to increase the tolerance to orthostatic stress [22].

PLR has been used to evaluate preload reserve extensively in the intensive care settings and shown to be accurate and useful in predicting fluid responsiveness [4], [7]. However, it has not been validated in pregnancy. To our knowledge, only one published study has evaluated fluid responsiveness in pregnant women using PLR [12] showing that it accurately predicts fluid responsiveness in oliguric women with severe pre-eclampsia with a sensitivity of 75% and specificity of 100%. Static measures of cardiovascular function are useful as threshold values, but functional parameters may be preferable for predicting disease as well as monitoring therapeutic interventions [2]. Preload reserve along with other functional hemodynamic parameters can be measured using simple non-invasive techniques. Whether they are useful in predicting pregnancy complications merits further investigation.

An increase in SV or CO or their surrogate, such as velocity time integral of sub-aortic blood flow measured by Doppler echocardiography following PLR has been commonly used as a predictor of fluid responsiveness. Although echocardiography is non-invasive, it has limitations related to operator-dependency. We used ICG as it is operator-independent, simple, non-invasive and ideally suited for serial measurement of changes over time [35]–[37]. Although there has been some doubt about the accuracy and applicability of this method in pregnancy [38],[39] and limitations have been highlighted [40], the hemodynamic measurements obtained using newer generation ICG machines have been validated and shown to be accurate [41],[42], reproducible, reliable and useful also in pregnant population [24],[43]–[45]. ICG has been demonstrated to have the ability to detect subtle changes in SV associated with change in maternal position [45].

In our study all participants were young, healthy women representing a normal population of reproductive age. The baseline characteristics of the study groups were similar except that the BMI was significantly higher in pregnant women compared to non-pregnant as expected. The actual mean weight of pregnant women was about 5 Kg higher compared to their booking weight. We chose not to use women's self-reported pre-pregnancy body weight because this information might be unreliable [46]. However, this is unlikely to have affected our results as we used indexed parameters of systemic blood flow and resistance while comparing differences between groups, and within group comparisons were performed using values obtained from the same individuals at baseline and PLR.

Non-pregnant women were examined at the follicular phase of their menstrual cycle to avoid variations in hemodynamic response caused by hormonal changes [47]. Pregnant women were examined at 22–24 weeks of gestation when the cardiovascular adaptive changes are fully established but the size of uterus is still unlikely to compromise venous return by the compression of inferior vena cava during PLR. Nevertheless, we have previously shown a good agreement between ICG measurements performed in supine semi-recumbent and left lateral positions [24]. For PLR we used leg elevation to 45° in association with trunk lowering from 45° semi-recumbent position to a flat horizontal position. Blood volume transferred to the central compartment is greater using this technique compared to PLR without trunk lowering due to the recruitment of venous reservoir of splanchnic organs in addition to lower extremities, and it is a preferred technique as it induces larger increase in cardiac preload [48].

Our study has some limitations. All study participants were white Europeans. Therefore, our findings may not be generalisable to multi-ethnic populations. Similarly, as the priori power calculation was performed assuming much larger effect size regarding preload responsiveness in pregnant women compared to what was actually observed later, the study may not have enough power to detect subtle differences between groups. To our knowledge, it is so far the largest study investigating maternal functional hemodynamics in normal pregnancy. However, gestational age associated serial changes in hemodynamic response to PLR cannot be inferred from this study due to the cross-sectional design. Whether response to PLR varies with gestational age needs to be further evaluated in a longitudinal study.

## Conclusions

Static measures of cardiovascular status are different between healthy pregnant and non-pregnant women, but the physiological response to PLR is similar and not modified by pregnancy at 22–24 weeks of gestation. Whether physiological response to PLR is different in earlier and later stages of pregnancy merit further investigation.
